# Endobiliary radiofrequency ablation for refractory cholangitis caused by mucin-producing intraductal papillary neoplasm of the bile duct

**DOI:** 10.1055/a-2878-2412

**Published:** 2026-07-22

**Authors:** Takahiro Urata, Shingo Ueno, Masato Kajiwara

**Affiliations:** 1 Department of Gastroenterology 38346 Japanese Red Cross Kumamoto HospitalKumamotoJapan

Mucin-producing intraductal papillary neoplasm of the bile duct (IPNB) is characterized by excessive mucin secretion and frequently causes refractory cholangitis 1 2 . Endoscopic management is often challenging, particularly in elderly patients unsuitable for surgical resection.

**Fig. 1:**
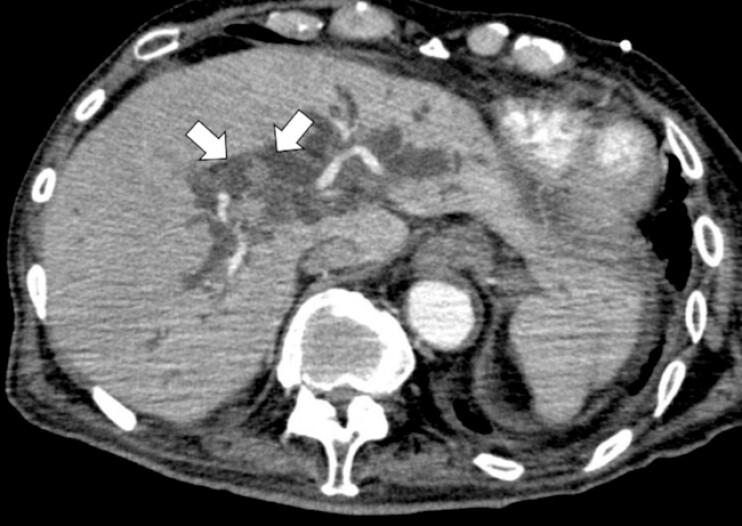
Contrast-enhanced CT showing a subtle lesion with faint enhancement at the orifice of the right anterior sectoral bile duct (arrow). CT, computed tomography.

**Fig. 2:**
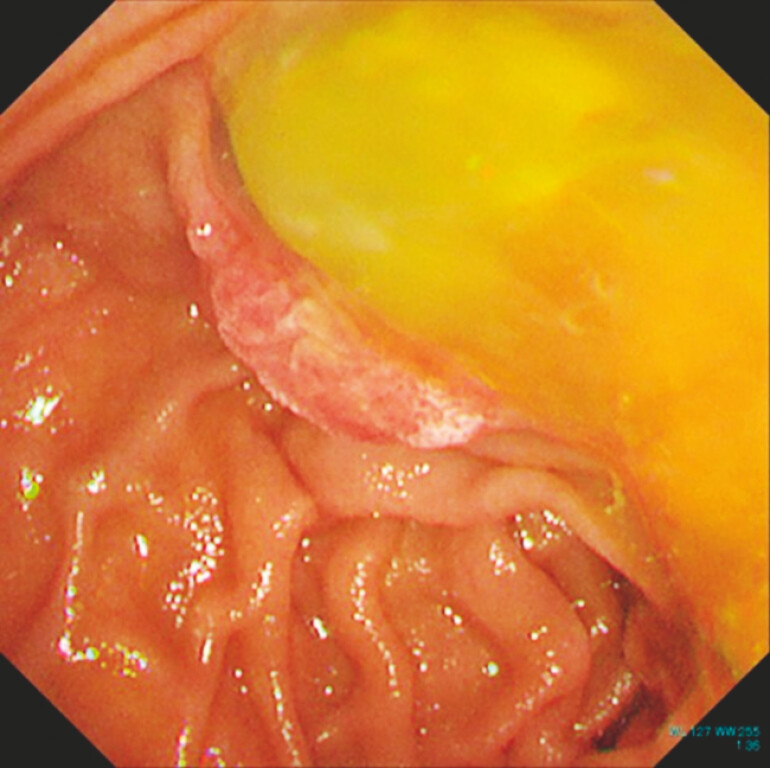
ERCP demonstrating a widely opened papilla with abundant mucin extrusion. ERCP, endoscopic retrograde cholangiopancreatography.

**Fig. 3:**
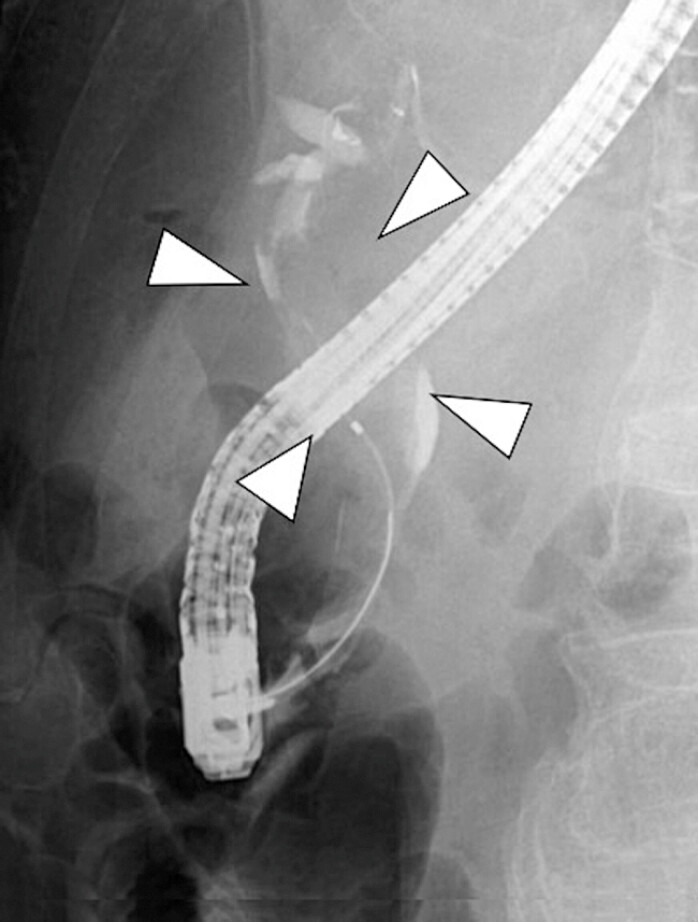
Peroral cholangioscopy revealing a mucin-producing papillary tumor near the hepatic hilum.

An 89-year-old man was referred to our hospital with recurrent episodes of acute cholangitis of unknown origin. Contrast-enhanced computed tomography revealed a subtle lesion with faint enhancement at the orifice of the right anterior sectoral bile duct ( Fig. 1 ). Endoscopic retrograde cholangiopancreatography demonstrated abundant mucin extrusion from a widely opened papilla ( Fig. 2 ) and multiple filling defects consistent with intraductal mucin. Peroral cholangioscopy (POCS) identified a mucin-producing papillary tumor near the hepatic hilum ( Fig. 3 ), and targeted biopsy confirmed the diagnosis of IPNB ( Video 1 ).

Peroral cholangioscopy-guided endobiliary radiofrequency ablation for the mucin-producing intraductal papillary neoplasm of the bile duct, with the confirmation of complete tumor ablation, the absence of mucin production, and good bile duct patency.Video 1

**Fig. 4:**
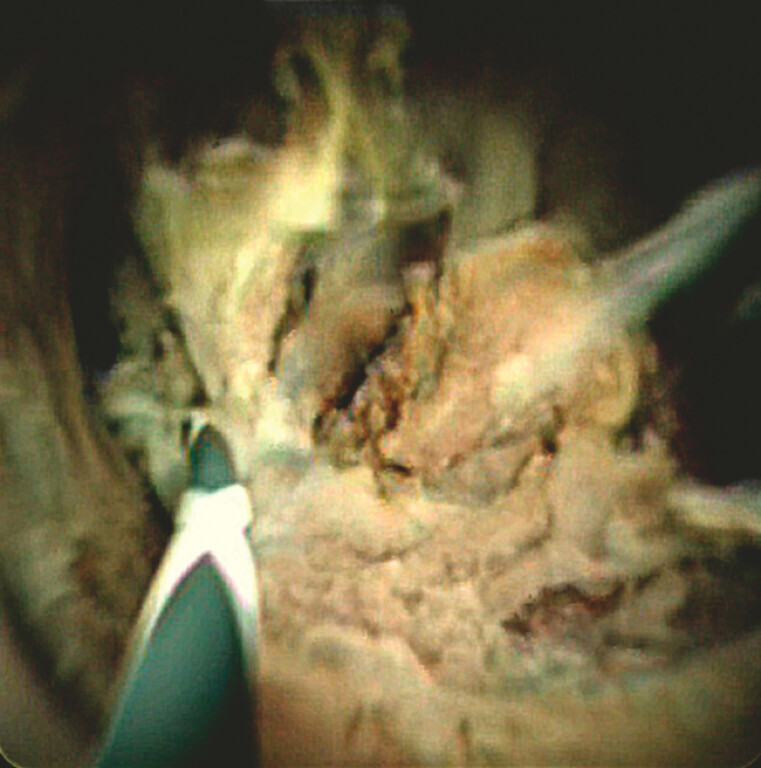
Peroral cholangioscopy immediately after radiofrequency ablation showing adequate ablation of the papillary tumor.

**Fig. 5:**
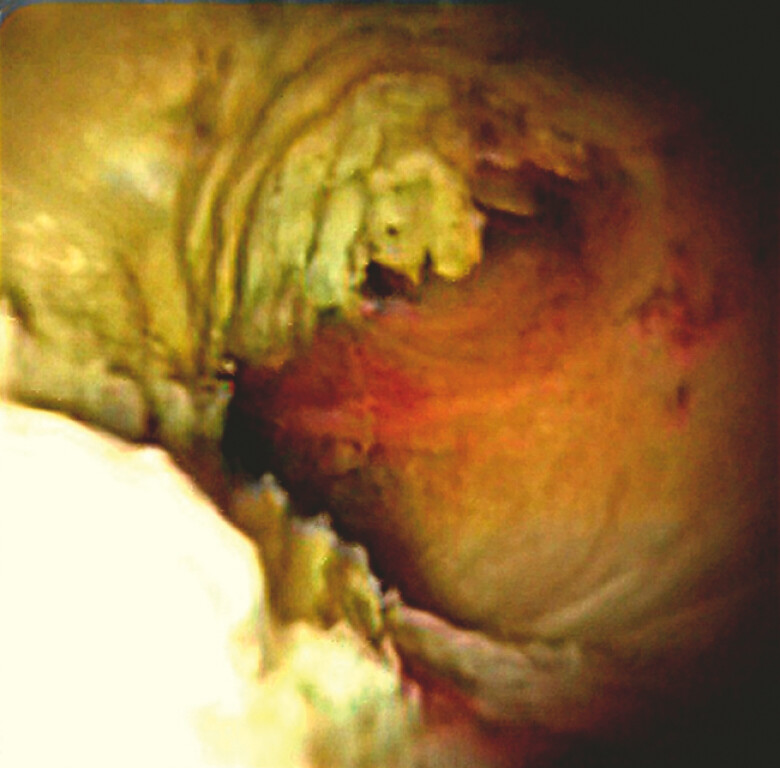
Peroral cholangioscopy after radiofrequency ablation demonstrating complete tumor ablation, the absence of intraductal mucin, and good bile duct patency.

Because of advanced age and multiple comorbidities, surgical resection was not indicated. Despite repeated biliary stenting, cholangitis recurred, suggesting insufficient control of mucin secretion. Therefore, palliative endobiliary radiofrequency ablation (RFA) was performed using a Habib EndoHPB catheter (Boston Scientific, Marlborough, Massachusetts, USA) under fluoroscopic guidance 3 4 . Two sessions were applied at 10 W for 90 seconds, aiming to reduce mucin-producing tumor tissue while minimizing bile duct injury. Immediately after RFA, POCS confirmed the effective ablation of the papillary tumor ( Fig. 4 , Video 1 ). Follow-up POCS 1 week later demonstrated complete tumor ablation, the absence of mucin production, and satisfactory bile duct patency ( Fig. 5 , Video 1 ). The biliary stent was removed. During 6 months of follow-up, no recurrence of cholangitis or procedure-related adverse events was observed.

This case highlights that carefully performed endobiliary RFA may represent a feasible and safe palliative option for controlling mucin secretion and preventing refractory cholangitis in patients with unresectable IPNB.

Endoscopy_UCTN_Code_CCL_1AZ_2AC

Citation Format

Endoscopy 2026; 58: E472–E473. DOI: 10.1055/a-2836-1486
